# Fatigue in Women with Fibromyalgia: A Gene-Physical Activity Interaction Study

**DOI:** 10.3390/jcm10091902

**Published:** 2021-04-28

**Authors:** Fernando Estévez-López, Diego F. Salazar-Tortosa, Daniel Camiletti-Moirón, Blanca Gavilán-Carrera, Virginia A. Aparicio, Pedro Acosta-Manzano, Víctor Segura-Jiménez, Inmaculada C. Álvarez-Gallardo, Ana Carbonell-Baeza, Diego Munguía-Izquierdo, Rinie Geenen, Eliana Lacerda, Manuel Delgado-Fernández, Luis J. Martínez-González, Jonatan R. Ruiz, María J. Álvarez-Cubero

**Affiliations:** 1 Department of Child and Adolescent Psychiatry/Psychology, Erasmus MC University Medical Center, 3015 GD Rotterdam, The Netherlands; fer@estevez-lopez.com; 2 Department of Ecology and Evolutionary Biology, University of Arizona, Tucson, AZ 85719, USA; 3 Department of Physical Education, Faculty of Education Sciences, University of Cádiz, 11519 Cádiz, Spain; daniel.camiletti@uca.es (D.C.-M.); bgavilan@ugr.es (B.G.-C.); victor.segura@uca.es (V.S.-J.); inma.alvarez@uca.es (I.C.Á.-G.); ana.carbonell@uca.es (A.C.-B.); 4 Department of Physiology, Faculty of Pharmacy, University of Granada, 18011 Granada, Spain; virginiaparicio@ugr.es; 5 Biomedical Research Centre (CIBM), Institute of Nutrition and Food Technology (INYTA), University of Granada, 18016 Granada, Spain; 6 Department of Physical Education and Sport, Faculty of Sport Sciences, University of Granada, 18010 Granada, Spain; acostapedro23@ugr.es (P.A.-M.); manueldf@ugr.es (M.D.-F.); 7 Physical Performance and Sports Research Center, Department of Sports and Computer Science, Section of Physical Education and Sports, Faculty of Sport Sciences, Universidad Pablo de Olavide, 41013 Seville, Spain; dmunizq@upo.es; 8 Department of Psychology, Faculty of Social and Behavioural Sciences, Utrecht University, 3508 TC Utrecht, The Netherlands; r.geenen@uu.nl; 9 Department of Clinical Research, Faculty of Infectious & Tropical Disease, London School of Hygiene & Tropical Medicine, London WC1E 7HT, UK; Eliana.Lacerda@lshtm.ac.uk; 10 GENYO, Centre for Genomics and Oncological Research, Pfizer, University of Granada, Andalusian Regional Government, PTS Granada, Av. Ilustracion, 114, 18016 Granada, Spain; luisjavier.martinez@genyo.es; 11 PROFITH—“PROmoting FITness and Health Through Physical Activity” Research Group, Department of Physical Education and Sport, Faculty of Sport Sciences, University of Granada, 18071 Granada, Spain; ruizj@ugr.es; 12 Department of Biochemistry and Molecular Biology III, Faculty of Medicine, University of Granada, 18010 Granada, Spain; mjesusac@ugr.es

**Keywords:** accelerometry, chronic pain, epidemiology, gene polymorphism, rehabilitation, treatment

## Abstract

Fatigue is a cardinal symptom in fibromyalgia. Fatigue is assumed to be the result of genetic susceptibility and environmental factors. We aimed at examining the role of genetic susceptibility for fatigue in southern Spanish women with fibromyalgia, by looking at single nucleotide polymorphisms in 34 fibromyalgia candidate-genes, at the interactions between genes, and at the gene-physical activity interactions. We extracted DNA from saliva of 276 fibromyalgia women to analyze gene-polymorphisms. Accelerometers registered physical activity and sedentary behavior. Fatigue was assessed with the Multidimensional Fatigue Inventory. Based on the Bonferroni’s and False Discovery Rate values, we found that the genotype of the rs4453709 polymorphism (sodium channel protein type 9 subunit alpha, *SCN9A*, gene) was related to reduced motivation (AT carriers showed the highest reduced motivation) and reduced activity (AA carriers showed the lowest reduced activity). Carriers of the heterozygous genotype of the rs1801133 (methylene tetrahydrofolate reductase, *MTHFR*, gene) or rs4597545 (*SCN9A* gene) polymorphisms who were physically active reported lower scores on fatigue compared to their inactive counterparts. Highly sedentary carriers of the homozygous genotype of the rs7607967 polymorphism (AA/GG genotype; *SCN9A* gene) presented more reduced activity (a dimension of fatigue) than those with lower levels of sedentary behavior. Collectively, findings from the present study suggest that the contribution of genetics and gene-physical activity interaction to fatigue in fibromyalgia is modest.

## 1. Introduction

Fibromyalgia is a disease characterized by chronic widespread pain and increased sensitivity to painful stimuli [1]. Although the pathophysiology of fibromyalgia remains unknown, family aggregation suggests a role of genetics in fibromyalgia [2]. Traditionally, most of the candidate-genes studied in fibromyalgia are related to neurotransmitters [3,4]. For instance, the most extensively studied gene is the catechol-*O*-methyltransferase (*COMT*), which participates in degrading catecholamines and several other neurotransmitters and, therefore, in modulating pain perception by the central nervous system. Findings of the previous literature regarding *COMT* gene and susceptibility to fibromyalgia are inconclusive [5,6], therefore, research has aimed at identifying new candidate genes; see [3,7]. For instance, the sodium voltage-gated channel alpha subunit 9 (*SCN9A*) gene have been recently proposed as part of the pathogenesis of fibromyalgia [8]. Mutations of the *SCN9A* gene may be related to upregulation of the sodium channels and, consequently, to hyperreactivity to nociceptive stimulus [9].

Currently, the diagnosis of fibromyalgia is undergoing some changes, and fatigue is now included as part of the clinical diagnostic criteria [10]. Indeed, fatigue is markedly high in fibromyalgia, e.g., it is severe in 82% of people with the disease [11]. People living with the disease identify fatigue as one of the main symptoms, with 25% of them identifying fatigue as the main symptom of the disease [12]. In fibromyalgia, fatigue is a distressing symptom that is associated with reduced health-related quality of life [13,14]. Most of the previous studies analyzed the association between candidate genes and a total score of symptoms of fibromyalgia (e.g., [15,16]); mostly using the total score of the Fibromyalgia Impact Questionnaire (FIQ). Unfortunately, this approach does not allow to study the determinants of specific key symptoms such as the levels of fatigue experienced by people with fibromyalgia. Collectively, previous findings suggest that the contribution of genetics to fibromyalgia is modest, which is expected because fibromyalgia is considered a very complex disease phenotype [7]. Therefore, it is hypothesized that the experience of fatigue in fibromyalgia is better understood as a genetic susceptibility that is modulated by environmental factors [4,17].

Epigenetic mechanisms represent a link between gene and environment [18] as it has been demonstrated in fibromyalgia [19] and similar diseases characterized by disabling fatigue (e.g., chronic fatigue syndrome) [20]. For instance, in people with chronic fatigue syndrome, increments in hypomethylation in promoter and regulatory regions of immune genes compared to other gene classes have been previously observed [20]. It has also been suggested that DNA methylation patterns in serum brain-derived neurotrophic factor (sBDNF) are key mechanisms explaining the pathophysiology of fibromyalgia and persistent fatigue [21]. Associations between fibromyalgia and single nucleotide polymorphism affecting the serotonergic, dopaminergic and catecholaminergic pathways have been identified via candidate gene analyses. Of them, those genes related to the serotonergic pathway and affecting ion channels are relevant in the levels of fatigue experienced by people with fibromyalgia [22]. Alteration of protein expression, potentially regulated by microRNA, may also play a role in fibromyalgia [23]. Indeed, it has been observed that alterations in microRNA are related to fatigue in people with fibromyalgia [24,25]. For instance, in comparisons to controls, the expression levels of miR-145-5p are reduced in fibromyalgia [25]. Additionally, lower levels of miR-145-5p expression are associated with more fatigue in fibromyalgia [25]. Therefore, gene-environment interactions may help to better understand the levels of fatigue in people with fibromyalgia.

When considering complex symptoms such as fatigue in fibromyalgia, gene-environmental interactions are likely present and can help to better understand the disease (e.g., unravel underlying mechanisms [26]). Among environmental exposures, physical activity (any bodily movement produced by skeletal muscles that results in energy expenditure above basal metabolic rate [27]) and sedentary behavior (activity performed while awake that is done in a seated or lying position and does not increase energy expenditure substantially [27]) are major determinants of health and disease [28]. Research has convincingly demonstrated that higher physical activity and lower sedentary behavior is beneficial for lowering fatigue in the general population [29] and diseases (e.g., cancer [30] and fibromyalgia [31,32]). Indeed, physical activity is a first-line non-pharmacological treatment for fibromyalgia symptoms [33] (including fatigue [31]). Despite the importance of physical activity and sedentary behavior in fibromyalgia, previous research did not consider the interplay of genes and these behaviors. From a clinical and public health perspective, to understand the interplay between genetics and physical activity on fatigue in fibromyalgia is of interest.

Therefore, this study aimed to comprehensively examine the singular association between genotype and fatigue, the interaction between genes, and the association between genes and physical activity/sedentary behavior with fatigue, which may lead to a better understanding of the biological and behavioral mechanisms of fatigue in people with fibromyalgia.

## 2. Materials and Methods

### 2.1. Study Design and Population

The design of the present study was cross-sectional. As described elsewhere [34], we calculated the sample size needed to obtain a representative sample of women with fibromyalgia from the Andalusian (Southern Spain) population, i.e., *n* = 240. Next, we followed a province-proportional recruitment strategy [34]. The participants (i) were recruited mainly via fibromyalgia associations from Andalusia (Southern Spain), (ii) had been previously diagnosed with fibromyalgia by a rheumatologist and met the 1990 American College of Rheumatology (ACR) criteria for fibromyalgia, which was further confirmed by a tender point examination [1], and (iii) signed an informed consent form. The Ethics Committee of the Virgen de las Nieves Hospital (Granada, Spain) approved the study (Registration number: 15/11/2013-N72). We followed the ethical guidelines of the Declaration of Helsinki.

### 2.2. Measures Related to Potential Confounders or Inclusion/Exclusion Criteria

Socio-demographic and clinical data. The participants filled out an initial questionnaire that included questions about date of birth, marital status, working status, and educational level, and presence/absence of acute or terminal illness (such as cancer, stroke, recent cardiomyopathy, severe coronary disease, schizophrenia, or any other disabling injury). The consumption of analgesics and antidepressants was registered as binary variables (yes/no).

Body fat (%) was measured using a portable eight-polar tactile-electrode impedanciometer (InBody R20, Biospace, Seoul, Korea). During the assessment, the participants were barefoot and they wore only underwear and no metal objects.

### 2.3. Measures Related to Genetic Analysis

As described elsewhere [4,35], samples were genotyped for 64 polymorphisms that had been previously investigated in relation to fibromyalgia susceptibility, symptoms, or potential mechanisms. We collected samples of buccal mucosa cells and performed DNA non-organic extraction (proteinase K and salting-out) procedures prior the spectrophotometric quantification (NanoDrop 2000c, ThermoFisher, Waltham, MA, USA). The data were analyzed using the TaqMan^®^Genotyper Software (ThermoFisher, Waltham, MA, USA) and downstream analysis using the AutoCaller™ Software.2.4 (ThermoFisher, Waltham, MA, USA).

### 2.4. Measures of Physical Activity and Sedentary Time

Triaxial accelerometers GT3X+ (Actigraph, Pensacola, FL, USA) were used to objectively measure physical activity and sedentary behavior during a time interval of seven continuous days with a minimum of 10 valid hours per day. Binary data of physical activity and sedentary behavior were recorded as follows (i) fulfilment (yes/no) of the physical activity recommendations (≥150 min/week of moderate to vigorous physical activity in bouts of, at least, 10 min of duration and, (ii) sedentary behavior (low/high) using the mean (459.1 min/day) as the cut-off value. Among the available brands, the Actigraph accelerometers (Actigraph, Pensacola, FL, USA) are clearly the most widely used in research [36,37]. Against doubly labelled water (the gold standard for measuring physical activity and sedentary behavior), the Actigraph (Actigraph, Pensacola, FL, USA) accelerometers are valid [38].

### 2.5. Measures Related to Fatigue

The Spanish version of the Multidimensional Fatigue Inventory (MFI) [39] was used to assess five dimensions of fatigue; namely, general fatigue (e.g., I feel tired), physical fatigue (e.g., physically I feel only able to do a little), mental fatigue (e.g., it takes a lot of effortto concentrate on things), reduced activity (e.g., I get little done), and reduced motivation (e.g., I don’t feel like doing anything). It should be noted that fatigue is by nature multidimensional, including physical and psychological domains [40,41]. The MFI accounts for this multidimensionality. The score ranges of the five dimensions is 4 to 20. The psychometric properties of the Spanish version of the MFI are adequate in people with fibromyalgia [39].

### 2.6. Statistical Analysis

All analyses were performed in the R environment v.3.4.1. The Hardy-Weinberg equilibrium (HWE; *p* > 0.01) and linkage disequilibrium (r^2^ > 0.5) were evaluated with ‘genetics’ package [42]. Gene-phenotype associations along with gene-gene interactions were assessed with the ‘SNPassoc’ package [43]. We developed our own script to study gene-environment interactions.

To analyze the singular associations of polymorphisms with phenotypes, we computed general linear models with age, body fat (%), and the consumption of analgesics and antidepressants as covariates. Interactions between polymorphisms of different genes as well as between polymorphisms and physical activity or sedentary behavior were assessed using the same models but including their interaction terms in separate models. We considered as significant those associations with either *p*-values lower than the Bonferroni’s correction or with both *p*-values and false discovery rate (FDR) values lower than 0.05.

## 3. Results

A total of 276 women participated in the present study. The participants have a mean age of 51.8 years (SD = 7.7), mostly are married (77.9%), and about half of them have finished primary school (50.9%). Furthermore, 36.2% of the participants were either on incapacity benefit, sick leave, or unemployed. The average sedentary behavior was 459.1 min/day (SD = 107.9), see Table 1.

The rs6323, rs7911, rs806377, rs1050450, rs1137070, rs3746544, rs4411417, and rs7124442 polymorphisms did not meet the HWE criteria. A low genotyping rate (i.e., ≤0.90) was observed for the rs4371369, rs4387806, rs6746030, rs7310505, rs9470080, and rs12620053 polymorphisms. The remaining 50 polymorphisms were included in the present study.

**Figure 1:**
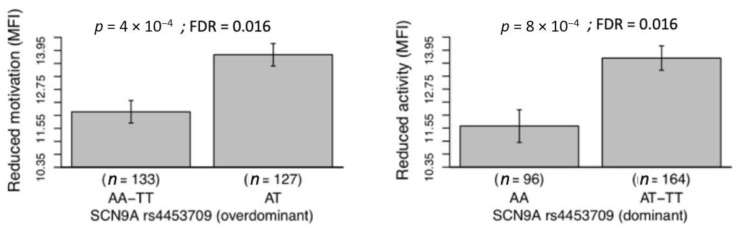
Individual associations of the genotype of the rs4453709 polymorphism (*SCN9A* gene) with dimensions of fatigue; i.e., reduced motivation (left panel) and reduced activity (right panel). *Note*. *SCN9A*, sodium voltage-gated channel alpha subunit 9 gene; MFI, multidimensional fatigue inventory (MFI, scores range 0–20). According to the *p*- and false discovery rate (FDR) values, all these associations of genotype and fatigue were significant.

Figure 1 shows that the AT genotype of the rs4453709 (*SCN9A* gene) showed that higher levels of reduced motivation than the AA/TT genotype (overdominant model, *p* = 0.0004 and FDR = 0.016) and that the genotype AA of the same polymorphism showed lower levels of reduced activity than the AT/TT genotype (dominant model, *p* = 0.0008 and FDR = 0.031). The remaining individual associations between genotype and fatigue outcomes were not significant (data not shown but available under reasonable request).

All the gene-gene interactions were not statically significant (data not shown but available under reasonable request).

**Figure 2:**
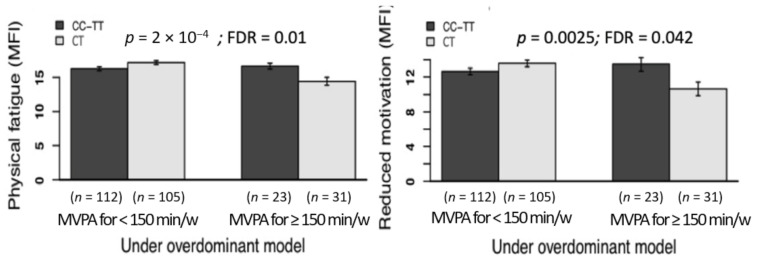
Associations of the interaction of the *MTHFR* gene (rs1801133 polymorphism)-physical activity with dimensions of fatigue; physical fatigue (left panel) and reduced motivation (right panel). *Note. MTHFR*, methylene tetrahydrofosfate reductase gene; MVPA, moderate to vigorous physical activity; MFI, multidimensional fatigue inventory (scores range 0–20). Physical activity was objectively measured using triaxial accelerometers GT3X+ (Actigraph, Pensacola, FL, USA). We dichotomized data of physical activity according to the fulfilment (yes vs. no) of the physical activity recommendations (≥150 min/week) of MVPA in bouts of, at least, 10 min of length. According to the *p*- and false discovery rate (FDR) values, all these gene-physical activity interactions were significant.

**Figure 3:**
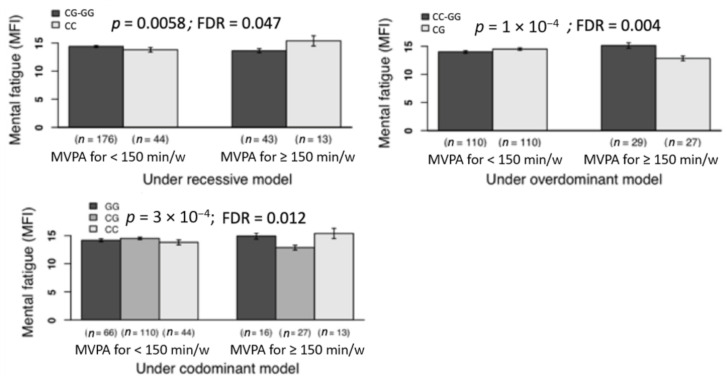
Associations of the interaction of the *SCN9A* gene (rs4597545 polymorphism)-physical activity with mental fatigue (a dimension of fatigue). *Note. SCN9A*, sodium voltage-gated channel alpha subunit 9 gene; MVPA, moderate to vigorous physical activity; MFI, multidimensional fatigue inventory (scores range 0–20). Physical activity was objectively measured using triaxial accelerometers GT3X+ (Actigraph, Pensacola, FL, USA). We dichotomized data of physical activity according to the fulfilment (yes vs. no) of the physical activity recommendations (≥150 min/week of MVPA in bouts of, at least, 10 min of length. According to the *p*- and false discovery rate (FDR) values, all these gene-physical activity interactions were significant.

A number of significant gene-physical activity interactions emerged. Figure 2 shows the significant interactions involving methylene tetrahydrofolate reductase (*MTHFR*) gene. Particularly, amongthe among carriers of the CT genotype of the rs1801133 (methylene tetrahydrofolate reductase, *MTHFR*, gene), those who met the physical activity recommendations showed lower physical fatigue and reduced motivation than those who did not meet such physical activity levels; *p* = 0.0002, FDR = 0.01 and *p* = 0.0025 and FDR = 0.042, respectively. Figure 3 shows the significant interactions involving the *SCN9A* gene. Particularly, in the participants carrying the CG genotype of the rs4597545 (*SCN9A* gene), in comparison to those who engage in low levels, the participants that had increased levels of physical activity reported lower mental fatigue, which was corroborated across different models: *p* = 0.0003 and FDR = 0.012 for codominant, *p* = 0.0058 and FDR = 0.047 for recessive, and *p* = 0.0001 and FDR = 0.004 for overdominant. A set of statistically significant additive associations were not interpreted as such given that they lacked statistical power (i.e., *n* ≤ 10 in some genotypes): of the rs1801133 (*MTHFR* gene) and physical activity with mental fatigue, and of the rs6860 (*CHMP1A* gene) and physical activity with mental fatigue, both under the recessive model. The remaining additive associations of genotype and physical activity with fatigue outcomes did not reach the significance (data not shown but available under reasonable request).

**Figure 4:**
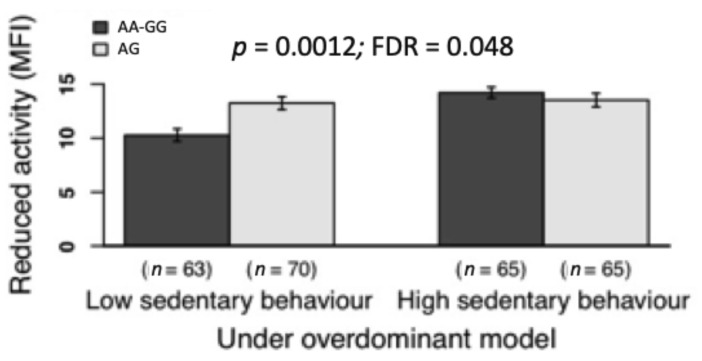
Associations of the interaction of the *SCN9A* gene (rs7607967 polymorphisms)-sedentary behavior with reduced activity (a dimension of fatigue). *Note. SCN9A*, sodium voltage-gated channel alpha subunit 9 gene; MFI, multidimensional fatigue inventory (scores range 0–20). Sedentary behavior as objectively measured using triaxial accelerometers GT3X+ (Actigraph, Pensacola, FL, USA). We dichotomized data of sedentary behavior (low vs. high) using the mean as the cut-off value. According to the *p* and false discovery rate (FDR) values, this gene-sedentary behavior interaction was significant.

Only a significant gene-sedentary behavior interaction emerged. Figure 4 shows that, in those participants carrying the AA/GG genotype of the rs7607967 (*SCN9A* gene), low sedentary behavior was associated with lower scores on the reduced activity dimension of the MFI (*p* = 0.0012, FDR = 0.048 for the overdominant model).

## 4. Discussion

The present study conducted in southern Spanish women with fibromyalgia showed that the genotype of the rs4453709 (*SCN9A* gene) was individually related to reduced motivation. Gene-gene interactions were not related to the phenotype of fatigue. We observed additive associations of the genotype (i) of the rs1801133 (*MTHFR* gene) and physical activity levels with physical fatigue and reduced motivation, (ii) of the rs4597545 (*SCN9A* gene) and physical activity levels with mental fatigue, and (iii) of the rs7607967 (*SCN9A* gene) and sedentary behavior with the reduced activity dimension of the fatigue phenotype. Therefore, the potential benefits of following an active lifestyle might be observed more clearly in women with fibromyalgia genetically predisposed to higher levels of fatigue.

It is widely accepted that a chronically sensitive central nervous system is part of the pathology of fibromyalgia [5,44]. It has been hypothesized that the dorsal root ganglion may also be hypersensitive to pain stimuli [9]. In the present study, we found that among the rs4453709 genotype (*SCN9A* gene), AT carriers reported the highest reduced motivation and AA carriers reported the lowest reduced activity. The *SCN9A* gene encodes a specific type of sodium channels (i.e., the Na(v)1.7) that are located highly in the dorsal horn of the spinal cord and in the dorsal root ganglion, the first structure is part of central nervous system while the latter receives afferent information from the peripheral nervous system. Thus, our findings may indicate a genetic vulnerability for reduced motivation and reduced activity (two dimensions of fatigue) in women with fibromyalgia involving a specific part of the central nervous system, which does not preclude a role of the peripheral one [45].

The Na(v)1.7 channels are crucial for pain signaling [46]. It is noteworthy that chronic pain and fatigue often co-exist in fibromyalgia [47] and their mechanistic pathways may be partly shared [48]. Moreover, the Na(v)1.7 channels are not exclusively related to pain, but also to other sensory stimuli such as acid sensing [49] and the cough reflex [50]. Therefore, our findings seem to extend the implication of the Na(v)1.7 channels from pain to fatigue in women with fibromyalgia. Sodium channels are key in the generation and conduction of action potentials. Thus, the *SCN9A* gene by modulating the Na(v)1.7 channels function might be especially involved in the experiencing of symptoms by people with fibromyalgia, particularly, reduced motivation and reduced activity. However, this speculation needs to be corroborated in future research.

The *MTHFR* gene encodes an enzyme that is central in the folate metabolism as a participant on the methionine-homocysteine cycle, which leads to DNA methylation [51]. The folate metabolism is key for feeding other biochemical cycles; its final product is an essential precursor for several neurotransmitters (e.g., serotonin) [52], some of them are related to fatigue (e.g., dopamine [53]). Previous literature suggested that the genotype of the rs1801133 (*MTHFR* gene) is associated with fatigue in people with migraine [54], and with stiffness and dryness in fibromyalgia [55]. Interestingly, in the present study the association of the rs1801133 (*MTHFR* gene) genotype and fatigue differed according to the physical activity levels of our participants. Additionally, metabolite abnormalities in the hippocampus of women with fibromyalgia are also related to the clinical picture of this disease [56]. It must be noted that the hippocampus is a core center in the appraisal of stress. In line with our findings, physical exercise improves the levels of metabolites [57] as well as the angiogenesis, neurogenesis, and connectivity of the hippocampus [58]. Therefore, physical activity might be particularly beneficial for women with fibromyalgia carrying specific genotypes. Further experimental research addressing this speculation is warranted.

Based on our findings, recommendations for future research may be considered. First, the most robust significant associations that emerged from the present study were for the (*SCN9A* and *MTHFR*) gene-physical activity interaction with the psychological domains of fatigue (i.e., reduced motivation and mental fatigue). Future studies testing whether these associations are replicated in an independent sample is warranted. Second, if they are replicated, attention should be paid to the magnitude of the association. In the present study, significant associations were modest and mostly for the psychological domains of fatigue. Third, further research analyzing other important symptoms (particularly, psychological or cognitive symptoms such as depression or difficulties performing cognitive tasks involving memory) of fibromyalgia is of interest to elucidate the relevance of the present findings in a wider context. Although the clinical relevance of the present findings is difficult to consider, it is worth considering that fatigue in fibromyalgia is a complex phenotype and, thus, fatigue is expected to be better explained by a combination of many small contributions, instead of large contributions of a few factors [59]. Finally, the European Alliance of Associations for Rheumatology (EULAR) indicated that physical exercise is the only therapy that has a strong level of evidence in fibromyalgia [33]. A meta-analysis of randomized controlled trials has determined that physical exercise has a moderate effect on reducing fatigue in fibromyalgia [31]. However, fibromyalgia is a heterogeneous population [60,61] and, currently, no treatment alternative has shown to be efficient, universally and in long-term in this population [31,33]. Additionally, there is large degree of variability in individual’s response to physical exercise even when they perform the same exercise protocol [62]. Therefore, it is of interest to determine, in future research, whether the genotype of people with fibromyalgia predicts the effectiveness of physical activity programs aimed at reducing the symptoms of the disease and, particularly, fatigue. This information may be of clinical relevance in the context of personalized medicine [63].

The findings of the present study should be considered in light of its limitations. Although unlikely given our large sample size and the inclusion of objective measurements of physical activity and sedentary behavior for 9 consecutive days, we welcome a replication study with an independent sample to test the robustness of the present findings. For some polymorphisms, our sample size was not large enough for testing the gene-people’s behavior’s interaction. On the other hand, we included a large number of candidate-genes, physical activity and sedentary behaviors were objectively measured for 7 consecutive days, and our results were adjusted for multiple comparisons. Although the most common approach in the past literature was to evaluate fatigue using unidimensional scales, the experience of fatigue is by nature multidimensional including physical and psychological domains (e.g., physically I feel only able to do a little and I don’t feel like doing anything, respectively) [40,41]. The inclusion of a multidimensional assessment of fatigue is also a strength of the present study.

## 5. Conclusions

In conclusion, we observed an association of the genotype of the rs4453709 polymorphism (*SCN9A* gene) with reduced motivation and reduced activity. We also found additive associations of the genotype (i) of the rs1801133 polymorphism (*MTHFR* gene) and physical activity levels with physical fatigue and reduced motivation, (ii) of the rs4597545 polymorphism (*SCN9A* gene) and physical activity levels with mental fatigue, and (iii) of the rs7607967 polymorphism (*SCN9A* gene) and sedentary behavior with the reduced activity dimension of the fatigue phenotype. Collectively, findings from the present study suggest that the contribution of genetics and gene-physical activity interaction to fatigue in fibromyalgia is modest.

## Figures and Tables

**Table 1:** Characteristics of the participants in the study, *n* = 276.

Clinical and Sociodemographic Information	*n* (%)	
Education Level		
Unfinished studies	26	(9.4)
Primary	139	(50.4)
Secondary (and vocational)	80	(29.0)
University	31	(11.2)
Marital status		
Married	215	(77.9)
Single	21	(7.6)
Separated/divorced	27	(9.8)
Widow	13	(4.7)
Working status		
Working	74	(26.8)
Household	93	(33.7)
Incapacity benefit or sick leave	55	(19.9)
Unemployed	45	(16.3)
Others	9	(3.3)
Medication		
Analgesics	247	(89.5)
Antidepressants	147	(53.3)
**Clinical and sociodemographic information**	**Mean (SD)**	
Age, years old	51.8	(7.7)
Body fat (%)	40.4	(7.6)
Tender points count (0–18)	16.9	(1.8)
Physical activity and sedentary behaviour		
Moderate-to-vigorous physical activity (min/week)	87.0	(119.2)
Sedentary behaviour (min/day)	459.1	(107.9)
Fatigue (MFI, 4–20)		
General fatigue	18.0	(2.5)
Physical fatigue	16.4	(3.1)
Reduced activity	12.8	(4.9)
Reduced motivation	12.9	(4.0)
Mental fatigue	14.7	(2.4)

SD, standard Deviation; MFI, multidimensional fatigue inventory.

## Data Availability

Data are available under reasonable request from F.E.-L., D.S.T., and M.D.-F.
